# Bromelain-based enzymatic burn debridement: Spanish multidisciplinary consensus

**DOI:** 10.1007/s00238-022-01999-2

**Published:** 2022-09-29

**Authors:** Jordi Serracanta, Jacinto Baena, José R. Martinez-Mendez, Manuel Sanchez-Sanchez, Eugenia Lopez-Suso, Rita Galeiras, Maria Dolores Perez-del-Caz, Carmen Vivo-Benlloch, Enrique Monclus-Fuertes, Jacobo Casalduero-Viu, Patricia Martin-Playa, Marta Ugalde-Gutierrez, Purificacion Gacto-Sanchez, Maria Dolores Rincon-Ferrari, Jose Maria Piqueras-Perez, Ana Martin-Luengo

**Affiliations:** 1 Department of Plastic Surgery and Burn Center, Vall d’Hebron University Hospital, Vall d’Hebron Barcelona Hospital Campus, Barcelona, Spain; 2 Department of Intensive Care and Burn Center, Vall d’Hebron University Hospital, Vall d’Hebron Barcelona Hospital Campus, Barcelona, Spain; 3 Department of Plastic Surgery and Burn Center, University Hospital La Paz, Madrid, Spain; 4 Department Intensive Care and Burn Center, University Hospital La Paz, Madrid, Spain; 5 Burn Unit, Complexo Hospitalario Universitario A Coruña, A Coruña, Spain; 6 Department of Plastic and Reconstructive Surgery and Burns, University Hospital La Fe, Valencia, Spain; 7 Department of Plastic and Reconstructive Surgery and Burns, University Hospital Miguel Servet, Saragossa, Spain; 8 Burn Unit, University Hospital Cruces, Bizkaia, Spain; 9 Burn Unit, University Hospital Virgen del Rocío, Seville, Spain; 10 Department of Plastic Surgery. Hospital, Universitario Rio Hortega de Valladolid, Valladolid, Spain; 11 Department of Intensive Care. Hospital, Universitario Rio Hortega de Valladolid, Valladolid, Spain

**Keywords:** Burn wound, Enzymatic debridement, Dermis preservation, NexoBrid**®**, Bromelain, Consensus, Delphi

## Abstract

**Background:**

Bromelain-based enzymatic debridement is gaining increased interest from burn specialists in the last few years. The objective of this manuscript is to update the previous, first Spanish consensus document from 2017 (Martínez-Méndez et al. 43:193–202, 2017), on the use of enzymatic debridement with NexoBrid**®** in burn injuries, adding the clinical experience of a larger panel of experts, integrating plastic surgeons, intensivists, and anesthesiologists.

**Methods:**

A consensus guideline was established by following a modified Delphi methodology of a 38-topic survey in two rounds of participation. Items were grouped in six domains: general indication, indication in critical patients, pain management, conditions for NexoBrid**®** application, NexoBrid**®** application technique, and post-debridement wound care.

**Results:**

In the first round, experts established consensus (strongly agree or agree) on 13 of the 38 statements. After the second round, a consensus was reached on 24 of the 25 remaining statements (97.2%).

**Conclusions:**

The present updated consensus document provides recommendations on the use of bromelain-based enzymatic debridement NexoBrid**®**, integrating the extensive clinical experience of plastic surgeons, intensivists, and anesthesiologists in Spain. Further clinical trials and studies are required to corroborate, modify, or fine tune the current statements.

**Level of evidence: Not ratable:**

## Introduction

Burns represent an important public health problem worldwide [1]. According to the World Health Organization, 180,000 deaths each year are caused by burns. Non-fatal burns are associated with temporary and permanent morbidities such as long and traumatic treatment, long hospitalization and post hospitalization care, final outcomes of disfigurement, and functional and psychological disabilities (including stigma and rejection) [1, 2]. Burn care depends on the patient’s general condition, the burn’s etiology, anatomic location, extent (frequently expressed as the percentage of total body surface area, TBSA), and depth, as well as associated comorbidities such as age or smoke inhalation [3]. The early removal of the burn eschar (or debridement) is one of the most important steps in the care of the deep partial and full thickness burns [4, 5]. Surgical, excisional debridement techniques are the current standard of care (SoC) for debridement in burn patients. However, these procedures have limitations such as significant trauma, major blood and heat loss, and potential damage to adjacent viable tissue that may be hard to differentiate from burned non-viable tissue [5]. Enzymatic debridement emerged as an alternative to cope with these limitations. It involves chemical or biological agents, including papain (from papaya) with urea, collagenases from *Clostridium histolyticum*, or the mixture fibrinolysin-desoxyribonuclease, that alone or in combination with surgical debridement help to remove the necrotic tissue (burn eschar) as a first stage of wound healing [2, 6]. However, currently available enzymatic agents are relatively slow and of modest efficacy [4]. In contrast, bromelain-based enzymatic debridement (NexoBrid**®**) has demonstrated to be highly effective, selective (removes burn eschar without harming viable tissues), and safe in deep partial-thickness and full-thickness burn wounds [4, 7, 8]. Effectively debriding burns early on admission has shown to reduce surgically related morbidities, blood and heat losses, infection rate, need for grafting, hospital stay, and costs, in comparison with SoC [8]. Since the approval of NexoBrid**®** in Europe in 2013, its use in burn centers has become more frequent [9]. In 2017, Martínez-Méndez et al. [10] published a consensus document on the use of enzymatic debridement with NexoBrid**®** in burn injuries in Spain. They involved a panel of seven experts (plastic surgeons) from major Spanish Burn Units. The objective of the present manuscript is to update the previous consensus document with additional clinical experience with bromelain-based enzymatic debridement from a larger panel of experts, integrating plastic surgeons, intensivists, and anesthesiologists.

## Methods

This consensus guideline paper has been created following a modified Delphi methodology [11], with two voting rounds. The first round was completed between October 14 and 19, 2020, and the second on October 23, 2020.

### Panelists

A total of 16 experts, representing eight of the major Burn Units in Spain (University Hospital Vall d’Hebron, Barcelona; University Hospital La Paz, Madrid; University Hospital Miguel Servet, Zaragoza; University Hospital La Fe, Valencia; Complexo Hospitalario Universitario A Coruña, A Coruña; University Hospital Cruces, Bizkaia; University Hospital Virgen del Rocío, Seville; and University Hospital Rio Ortega, Valladolid), were invited to participate in the study. A prerequisite for every participant was vast experience in the use of NexoBrid**®** (all panelist have experience using Nexobrid for the last 5 years, adding up more than 1000 cases treated by the entire group of participants). Of this group, 15 (eight plastic surgeons, six intensivists, and one anesthesiologist) completed the survey and finalized the consensus paper. There are 9 certified burn centers in Spain, and only one did not participate in the consensus. The 8 burn centers which took part in the consensus treat the vast majority of burn patients in Spain. Moreover, almost the totality of the enzymatic debridements has been performed in this 8 burn units, so that all the centers that have experience in the use of Nexobrid are represented in this consensus eliminating the risk of bias.

These units that use Nexobrid base their work on the recommendations of the technical data sheet of the manufacturer’s product, on-site training performed by the producer peer-reviewed publications and the AEMPS (Spanish Agency for Medicines and Medical Devices) guidelines, so that the consensus recommendations are congruent and compatible with those given by the manufacturer and the AEMPS as well as the wealth of information by other specialists.

### Survey

A 38-item survey was created based on the previous consensus document on the use of NexoBrid**®** in Spain [10], adding new topics from other guidelines [5]. Topics were grouped in six domains: general indications (items 1 to 10), indications in critical patients (items 11 to 17), pain management (items 18 to 21), the conditions for NexoBrid**®** application (items 22 to 27), technique of application (items 28 to 34), and post-debridement care (items 35 to 38). The degree of agreement or disagreement with the consensus statements was determined by using a 5-point Likert scale. Aside from six statements (14, 15, 16, 17, 37, and 38), all others were evaluated quantitatively with a Likert scale varying from 1 (strongly disagree), 2 (disagree), 3 (neither agree nor disagree), 4 (agree), to 5 (strongly agree). In these six topics, five possible statements were offered to be chosen or ordered, when appropriate. All 38 questions of the survey are based on those used in the Spanish consensus of 2017 adding new questions proposed in other papers.

### Delphi rounds and agreements

During the first round, all experts anonymously completed the survey. The survey was provided to the experts via Google Forms by the coordinating group, and subsequently, the results were compiled (percentage of each response for each item). The second round consisted on online web meeting, where participating experts reviewed the results of the first round and discussed the interest and details of each statement. Consensus was established when all the participants totally or strongly agreed with each statement. In some cases, the statement had to be modified (rewritten) in order to provide a more direct and clear statement for readers.

## Results

### First round

Experts established consensus (strongly agree or agree) on 13 statements (1, 2, 10, 11, 18, 20, 21, 25, 28, 33, 34, 35, and 36), whereas they showed different opinions on 25 statements (topics 3, 4, 5, 6, 7, 8, 9, 12, 13, 14, 15, 16, 17, 19, 22, 23, 24, 26, 27, 29, 30, 31, 32, 37, and 38). Opinions given during the first round, with the original statements, are shown in Table 1.

**Table 1:** Opinions shown in the first round

Topic	Domain	Statement	Results (%)
1	General indication	The enzymatic debridement is not indicated for epidermal or superficial dermal burns, whereas it can be used in other degrees of burn	SA: 73.3 A: 26.7
2		The enzymatic debridement for the treatment of burns should only be used by experienced professionals after adequate training	SA: 86.7 A: 13.3
3		The enzymatic debridement is a safe tool for the removal of the eschar in adult patients and it can be safely used by following the Summary of Product Characteristics	SA: 86.7 NAND: 6.7 D: 6.7
4		The enzymatic debridement can be used in pediatric patients with satisfactory results, but currently it is off-label	SA: 53.3 A: 26.7 NAND: 13.3 D: 6.7
5		The use of the enzymatic debridement can be very beneficial for the treatment of face, hands, neck, and neckline by saving vital dermal tissue; it is very useful in the treatment of thorax and abdomen for bleeding reduction	SA: 60.0 A: 33.3 D: 6.7
6		The clinical evaluation of burn depth is a sufficient indication for treating with enzymatic debridement	SA: 53.3 A: 40.0 D: 6.7
7		The enzymatic debridement can be safely used in a single application on the anatomical area up to 15% TBSA, but there are data that indicate that sequential-deferred applications up to 15% TBSA on different areas are safe	SA: 64.3 A: 28.6 D: 7.1
8		After the first application, it is possible to applicate the enzymatic debridement in the same patient on different anatomical areas during the following days	SA: 60.0 A: 33.3 D: 6.7
9		The main indication for enzymatic debridement is the eschar removal in thermal burns (flame, scalds, contact); its use is not recommended for the chemical and electrical burns	SA: 53.3 A: 40.0 D: 6.7
10		The enzymatic debridement is useful in the early removal of the eschar in circumferential burns of extremities: It has demonstrated to reduce the need of surgical escharotomies in these patients	SA: 93.3 A: 6.7
11	Indication in critically ill patients	Enzymatic debridement can increase the systemic inflammatory response in critical patients and, in some cases, cause transient hemodynamic instability	SA: 73.3 A: 26.7
12		The use of enzymatic debridement requires a previous hemodynamically stabilization	SA: 46.7 A: 33.3 NAND: 20.0
13		Hypovolemia should be corrected before applying the enzymatic debridement	SA: 93.3 NAND: 6.7
14		The application of enzymatic debridement in critical patients is safe but it can require close monitoring (order the responses)	Hemodynamic: 71.4 Nursing: 14.3 Medical: 14.3
15		In critical patients, NexoBrid**®** should not be used within the first hours, until after the phase of maximum permeability alteration (except in circumferential burns where the compartment syndrome can be prevented). What time do you consider appropriate to start the enzymatic debridement?	6–12 h: 6.7 12–24 h: 20.0 After 24 h: 13.3 Whenever, if clinically stable: 60.0
16		In critical patients, enzymatic debridement should be used in a sequential manner, depending on the response of the patient. Applying it to surfaces lower than 15% and repeating the process as soon as the hemodynamic situation of the patient allows it, and as many times as necessary. Which are the most frequent challenges to deal with?	No problems: 13.3 Hemodynamic instability: 20.0 Coagulation alterations: 20.0 Human resources: 40.0 Others: 6.7
17		Do you consider some evaluable cases for the off-label use of NexoBrid**®** in more than 15% TBSA in a single application?	Just if compartment syndrome in more than one extremity: 46.7 In critical patients to whom early surgery may not be available: 33.3 Never: 6.7 Others: 13.3
18	Pain management	The adequate management of pain is needed in all steps of debridement	SA: 93.3 A: 6.7
19		In adult patients, the utilization of intravenous sedoanalgesia is safe and effective during the application and removal of NexoBrid**®**, being enough an analgesia during the hours of debridement and both opioids and non-opioids could be used effectively (EVA < 3)*	SA: 52.9 A: 11.8 NAND: 11.8 D: 23.5
20		Locoregional anesthesia is an alternative to intravenous sedoanalgesia in patients with burns in extremities. In adult patients, enzymatic debridement does not require, routinely, general anesthesia*	SA: 70.6 A: 29.4
21		The procedure does not require an operating room and it can be performed with adequate resources of the expert personnel (anesthesiologist or intensivists) and monitoring at the patient’s bed*	SA: 94.1 A: 5.9
22	Conditions for NexoBrid**®** application	The enzymatic debridement can be used immediately after the clinical evaluation of burn depth and wound cleansing: the removal of blisters and keratin remnants is necessary before its application	SA: 53.3 A: 33.3 NAND: 6.7 D: 6.7
23		In the early use of the enzymatic debridement (within 72 h of injury), the standard burn cleansing and saline washing immediately before the application of a presoaking (wet dressing) of at least 2 h is needed for an effective debridement	SA: 26.7 A: 13.3 NAND: 33.3 D: 26.7
24		The enzymatic debridement can be applied up to 5 days post injury in presence of wet eschars: the delayed application requires an adequate preparation of the burn wound by mechanical removal of superficial layers of charred/desiccated tissues and wet dressing	SA: 53.3 A: 33.3 NAND: 13.3
25		The use of an antiseptic solution is needed in both early and delayed applications in presence of contaminated or infected wounds	SA: 86.7 A: 13.3
26		The use of enzymatic debridement is not recommended in case of clinical evidence of infection	SA: 46.7 A: 13.3 NAND: 40.0
27		The coagulopathy must be corrected before the application of enzymatic debridement	SA: 66.7 A: 6.6 NAND: 26.7
28	Application technique for NexoBrid**®**	The enzymatic agent must be applied for approximately 4 h	SA: 53.3 A: 46.7
29		In adult and pediatric patients, the recommended application implies the use of approximately 2 g for 1% TBSA or 180cm^2^, or about a 3 mm thick layer	SA: 28.6 A: 64.3 D: 7.1
30		The standard application of the drug (direct spreading over the wound and delimitation with a physical barrier of vaseline), can be optimized in terms of ease of use and surface contact with the burned area by first distributing the drug on a non-stick gauze in order to concentrate it more evenly on the lesion	SA: 30.8 A: 23.1 NAND: 23.1 D: 23.1
31		At the end of the phase of enzymatic debridement, a thorough manual cleansing should be performed for the removal of dissolved tissue and product remnants	SA: 46.7 A: 20.0 D: 33.3
32		The use of wet dressing/soaking for 2–28 h is indicated for complete removal of dissolved eschar and the remnants of the product	SA: 46.7 A: 40.0 D: 6.7 SD: 6.7
33		The color of the wound bed and the bleeding pattern after moist dressing can help to confirm the clinical evaluation of burn depth	SA: 73.3 A: 26.7
34		The enzymatic debridement dramatically reduces blood loss in comparison with surgical treatment	SA: 53.3 A: 46.7
35	Post-debridement wound care	After the enzymatic debridement, in presence of residual dermal tissue, wet dressings that maintain a moist environment are the optimal dressing to facilitate the spontaneous healing, alternatively to the homologous skin graft	SA: 71.4 A: 28.6
36		After an effective enzymatic debridement, in absence of viable dermal tissue, a definitive coverage and wound closure must be performed with an autograft, after adequate preparation of the wound bed	SA: 80.0 A: 20.0
37		In case of autografts, when would you perform it?	As soon as possible: 14.3 24 h: 14.3 48–72 h: 14.3 24 h: 14.3 3–5 days: 42.9 After 5 days: 14.3
38		In case of a delayed autograft is indicated for a non-epithelializing post NexoBrid**®** wound be, how low would you wait to make the decision d?	Less than 21 days: 35.7 21–30 days: 50.0 30–50 days: 7.2 Wait until epithelizes, despite being slow: 7.1

*SA* strongly agree, *A* agree, *NAND* neither agree nor disagree, *D* disagree, *SD* strongly disagree

^*^These statements for pain management were responded by 17 participants

### Second round: final statements

Experts reached consensus on 24 of the 25 remaining statements after the second round. No consensus was met for statement 19 regarding pain management. Moreover, 20 statements were modified (4, 5, 6, 9, 11, 12, 14, 15, 16, 17, 19, 20, 22, 27, 29, 32, 33, 34, 37, and 38). Experts recommended not to include statements 26 and 30 among the final ones because, in their opinion, these could be misunderstood and did not provide an added value to this consensus. Therefore, consensus was met in 35 out of 36 items (97.2%).

A recent article published by Niederberger and Spranger [12] performed a systematic review of the methods used in 12 different health-related Delphi studies. As stated in the article, a large number of modifications to the Delphi technique have been developed. Usually, these studies include a deliberately selected panel of experts and are carried out in 2–3 rounds, thus allowing discussions among the panelists in order to modify consensus statements as needed.

The following are the final statements:

#### Agreement on general indication

1. *“Enzymatic debridement is indicated for deep dermal burns. It would not make any contribution to the healing process in first degree burns.”*
2. *“The enzymatic debridement for the treatment of burns should only be used by experienced professionals after adequate training.”*
3. *“The enzymatic debridement is a safe tool for the removal of the eschar in adult patients and it can be safely used by following the Summary of Product Characteristics.”*
4. *“The use of enzymatic debridement in pediatric patients is considered an off-label use and provides results comparable to those in adults in specific cases. Our results support several peer reviewed publications, including other consensus guidelines.”*
5. *“The use of the enzymatic debridement can be very useful for the treatment of face, hands, neck, and neckline by saving vital dermal tissue; it is very useful in the treatment of thorax and abdomen for bleeding reduction in compared to tangential debridement.”*
6. *“The clinical evaluation of burn depth performed by an expert is a sufficient indication for treating with enzymatic debridement.”*
7. *“The enzymatic debridement can be safely used in a single application on anatomical areas up to 15% TBSA, but there are data that indicate that sequential, deferred applications up to 15% TBSA on different areas are safe.”*
8. *“After the first application, it is possible to apply the enzymatic debridement in the same patient on different anatomical areas during the following days.”*
9. *“The main indication for enzymatic debridement is the eschar removal in thermal burns (flame, scald, contact), there is not enough information for its use on chemical and high-voltage electrical burns.”*
10. *“Since enzymatic debridement can be applied during the first hours after the burn injury, it can avoid the need for surgical escharotomies in patients with circumferential burns of the extremities, with or prone to develop BICS (burn induced compartment syndrome).”*

#### Agreement on the indication for critically ill patients

11) *“Enzymatic debridement can increase the systemic inflammatory response in critical patients and, in some cases, cause hemodynamic instability.”*
12) *“The use of enzymatic debridement requires a hemodynamically stabilized patient.”*
13) *“Hypovolemia should be corrected before applying the enzymatic debridement.”*
14) *“The application of enzymatic debridement in critical patients is safe but requires close monitoring and increased workload for the medical and nursing teams.”*
15) *“In critically ill patients, NexoBrid® should not be used until the patient has been properly stabilized, except in circumferential burns where its early use will prevent the development of compartment syndrome and avoid the need for escharotomy.”*
16) *“In critical patients, enzymatic debridement should be used in a sequential-deferred manner, depending on the response of the patient, by applying it to surfaces lower than 15% of TBSA and repeating the process as soon as the hemodynamic situation of the patient allows it, and as many times as necessary. The challenges that experts more frequently have to deal with are related to human resources and coagulation alterations.”*
17) *“Off-label use of NexoBrid® in more than 15% of TBSA in a single application could only be considered in cases of circumferential burns on more than one limb to prevent development of compartment syndrome, and in critically ill patients for whom early surgery may not be possible (e.g., mass casualty incidents).”*

#### Agreement on pain management

18) *“The adequate management of pain is needed in all steps of debridement.”*
19) *“The pain management strategy during the enzymatic debridement can change taking into account the type of the patient, the resources, and the experience of each working group. Regarding this, most of the participating centers prefer to use intravenous sedoanalgesia during the application and removal of NexoBrid****®****, and analgesia during the hours of debridement.”*
20) *“Locoregional anesthesia is a widely used alternative to intravenous sedoanalgesia in patients with burns affecting extremities. The enzymatic debridement does not routinely require general anesthesia.”*
21) *“The procedure does not require an operating room and it can be performed with appropriated staff (anesthesiologist or intensivists) and bed-side monitoring.”*

#### Agreement on the conditions for NexoBrid® application

22) *“The enzymatic debridement can be used after the clinical evaluation of burn depth and wound cleansing: the removal of blisters and keratin remnants is necessary before its application.”*
23) *“In the early use of the enzymatic debridement (within 72 h of injury), a standard burn cleansing and saline washing performed immediately before the application of a presoaking (wet dressing) of at least 2 h are needed for an effective debridement.”*
24) *“The enzymatic debridement can be applied up to 5 days post injury as long as the scar is still wet: delayed applications require an adequate preparation of the burn wound by mechanical removal of superficial layers of charred/desiccated tissues, and wet dressing.”*
25) *“The use of an antiseptic solution is needed in both, early and delayed application when the wound is contaminated or infected.”*
26) *“Any coagulopathy should be corrected before the application of enzymatic debridement.”*

#### Agreement on the application technique for NexoBrid®

28) *“The enzymatic agent must be applied for approximately 4 h.”*
29) *“The recommended application implies the use of approximately 2 g for 1% TBSA of an adult or 180cm*^*2*^*, or about a 3 mm thick layer.”*
30) *“At the end of the phase of enzymatic debridement, a thorough manual cleansing should be performed for the removal of debrided tissue and dissolved product remnants.”*
31) *“The use of wet dressing/soaking for 12–24 h is indicated for complete removal of debrided eschar and the remnants of the product.”*
32) *“The color of the wound bed and the bleeding pattern after the removal of the product are useful to confirm the clinical evaluation of burn depth.”*
33) *“The enzymatic debridement reduces blood loss compared to tangential excisional debridement.”*

#### Agreement on wound care after debridement

35) *“After the enzymatic debridement, when there is residual dermal tissue, a dressing that maintains a moist environment to facilitate spontaneous healing is needed. There is a variety of options such as: Suprathel****®****, Mepilex Ag****®****, Biobrane****®****, or homografts.”*
36) *“After an effective enzymatic debridement, in absence of viable dermal tissue, a dressing with Mepilex Ag****®**** is applied until the wound is grafted. Prior to the autograft the wound bed is usually prepared with Versajet****®**** or surgical brush.”*
37) *“If autografts are indicated, they should be performed between day 3 and day 5 after enzymatic debridement.”*
38) *“If a delayed autograft is indicated for a not healing post NexoBrid****®**** wound bed, it is recommended to wait between 21 and 30 days for this decision.”*

## Discussion

Bromelain-based enzymatic debridement has gained increasing attention from burn specialists in recent years [9, 13]. NexoBrid**®** is indicated for the removal of the eschar in adults with deep partial- and full-thickness thermal burns [14]. According to the Summary of Product Characteristics (SmPC), it should not be used in more than 15% TBSA per session. NexoBrid**®** is especially useful in risky anatomical locations and those with underlying functional structures [4]. Indeed, enzymatic debridement has shown to be superior regarding tissue preservation, completeness of debridement, and wound closure compared to SoC when applied to hand and face [8, 15]. Enzymatic debridement is also effective for the avoidance of surgical escharotomy in circumferential deep burns of the distal upper extremity [9, 16]. Compared to SoC, NexoBrid**®** significantly reduces debridement-related blood loss [4]. Nevertheless, the risk of bleeding can increase with anticoagulant therapies or coagulation abnormalities, present in up to 40% of critical patients [5, 17]. NexoBrid**®** application time should not exceed 4 h because its activity decreases [18]. Pre-treatment before NexoBrid**®** with agents containing silver, iodine, and copper should be avoided as they inhibit bromelain activity. Presoaking with water-based solutions such as polyhexanide-containing agents is recommended [19]. NexoBrid**®** application is painful during the first 45–60 min, described as burning sensation [20] and necessitating adequate pain management, with analgesia and/or anesthesia. Recently, two consensus guidelines on the use of the bromelain-based enzymatic debridement have been published [5, 9]. Hirche et al. [9] updated the European consensus guidelines established in 2017 with the experience of a multiprofessional panel of plastic surgeons and burn care specialists from 12 European Burn Centers. They not only reviewed similar topics as in the present document (such as indications, pain management, post-debridement care, NexoBrid**®** application, and technique); but also included new topics related to the timing of application, skin grafting, outcomes, cost-effectiveness, patient’s perspective, logistic aspects, and training strategies [9]. They met consensus on 42 out of 43 statements. The only statement with no consensus was regarding the ambulatory care with NexoBrid**®** in minor burns. Ranno et al. [5] carried out a consensus guideline for Italian NexoBrid**®** users. Their 27-item survey comprised the domains of indications, pain management, application timing and technique, and post-enzymatic debridement wound care. The panel of 14 experts established consensus on 26 of the statements. The only statement that did not reach strong agreement was related to the use of NexoBrid**®** in pediatric population. Similar to our study, the fact that its use in pediatric patients is considered off-label and the lack of experience were the reasons for not meeting an agreement. At the time of writing the present manuscript, the use of NexoBrid**®** is considered off-label in patients under 18 years old, and most of our participating experts have no experience in this population. Nevertheless, those who did use NexoBrid**®** in children (3 centers) achieved successful results with no major complications. Scientific evidence is required to safely use NexoBrid**®** in pediatric population. The published literature addressing this issue is scarce. Shoham et al. [21] reviewed the experience of NexoBrid**®** in three clinical trials with 110 children. Of them, 77 received treatment with NexoBrid**®** in a phase I/II trial, and achieved complete eschar removal in 92.7% of treated areas, within 0.9 days from hospital admission. The remaining 33 children participated in a phase III randomized clinical trial (17 were treated with NexoBrid**®** and 16 with SoC). Complete eschar removal was reported in all individuals receiving NexoBrid**®** in 0.9 days from initiation (versus 6.5 days in SoC). An additional randomized controlled pediatric phase III clinical trial is currently ongoing (clinicaltrials#NCT02278718), evaluating the efficacy and safety of NexoBrid**®** in children with thermal burns compared with SoC. Another topic that could be considered off-label is the use of NexoBrid**®** on more than 15% TBSA. In the Italian consensus guideline [5], like ours, most of the experts state they have used NexoBrid**®** in a sequential-deferred manner, 15% TBSA sessions on different areas, and even up to 20–25% per session on the same patient with large burn areas. Effective enzymatic debridement of burn wounds depends on the denaturation status of collagen [22].

Our present consensus on the use of NexoBrid**®** represents an update of the previous document [10], with clinical experience from a multidisciplinary panel of experts providing user-oriented recommendations aiming to optimize its adequate application for inexperienced healthcare professionals. The presence of six intensivists and one anesthesiologist provided great insight of some aspects such as pain management, fluid therapy, and coagulation, and represent an added value regarding the previous consensus [10]. Our statements corroborate the European and Italian consensus guidelines [5, 9]. However, our document also integrates the experience of both plastic surgeons and intensivists, who are the main expert professionals using the enzymatic debridement in Burn Centers in Spain. This multidisciplinary teamwork allowed to apply treatment with Nexobrid**®** to patients affected by Covid-19 [23]. Moreover, in this document, we provided clarified consensus statements in those topics with no clear initial agreement, instead of presenting a percentage of consensus (as done in the first round Table 1). By following this procedure, the less experienced reader may gain insight from all statements.

### Limitations

The main limitation of any consensus document is potentially rooted in its intrinsic subjective nature. Experts provide their professional experience in clinical practice. Therefore, statements cannot be considered asa scientifically based, definitive evidence. Further clinical trials and long-term clinical studies are required to corroborate or modify the current statements.

## Conclusion

The present updated consensus document provides recommendations on the use of bromelain-based enzymatic debridement with NexoBrid**®**, merging the extensive clinical experience of plastic surgeons and intensivists in Spain.
